# The Use of Virtual Reality for Pain and Anxiety Reduction: A Literature Review

**DOI:** 10.15388/Amed.2026.33.1.2

**Published:** 2026-07-22

**Authors:** Gerda Marčiūtė, Ignas Lapeikis, Greta Kasputytė

**Affiliations:** 1 Lithuanian University of Health Sciences, Faculty of Medicine E-mail: ORCID ID; 2 Lithuanian University of Health Sciences, Faculty of Medicine E-mail: ORCID ID; 3 Lithuanian University of Health Sciences, Medical Academy, Faculty of Medicine, Department of Anaesthesiology; Lithuanian University of Health Sciences, Simulation Centre of Medical Academy E-mail: ORCID ID

**Keywords:** virtual reality, immersive VR, procedural pain, anxiety, interventions, virtualioji realybė, imersinė virtualioji realybė, virtualioji aplinka, procedūrinis skausmas, nerimas

## Abstract

Virtual reality (VR) is being increasingly used as an adjunctive tool to reduce procedural pain and anxiety during various interventional procedures. Forty articles published between 2020 and 2025 were reviewed. The articles reviewed discuss the use of virtual reality to reduce procedural pain and anxiety during various interventional procedures. The results of the literature analysis show that VR can effectively direct patients’ attention and reduce the subjective intensity of pain and fear during procedures across different age groups. VR has been successfully applied to interventional procedures such as vaccination, blood draws, and peripheral vein punctures in children, as well as to a wide range of interventions in obstetrics, orthopaedics, endovascular surgery, cardiology, and dentistry. The studies reviewed confirm that VR is a safe method that rarely causes adverse side effects and can be integrated into clinical practice, contributing to patient comfort, emotional well-being, and the overall satisfaction with the procedure.

## Abstract (it)

Virtualioji realybė (VR) vis dažniau taikoma kaip papildoma priemonė procedūriniam skausmui ir nerimui mažinti atliekant įvairias intervencines procedūras. Buvo apžvelgta keturiasdešimt 2020–2025 m. publikuotų straipsnių. Apžvelgtuose straipsniuose aptariamas virtualiosios realybės taikymas siekiant sumažinti procedūrinį skausmą ir nerimą įvairių intervencinių procedūrų metu. Literatūros analizės rezultatai rodo, kad VR gali veiksmingai nukreipti įvairių amžiaus grupių pacientų dėmesį ir sumažinti subjektyviai patiriamo skausmo bei baimės intensyvumą procedūrų metu. VR sėkmingai taikyta atliekant tokias intervencines procedūras, kaip antai vakcinacija, kraujo ėmimas ir periferinių venų punkcija vaikams, taip pat įvairias intervencijas akušerijoje, ortopedijoje, endovaskulinėje chirurgijoje, kardiologijoje ir odontologijoje. Apžvelgti tyrimai patvirtina, kad VR yra saugus metodas, retai sukeliantis nepageidaujamą šalutinį poveikį ir galintis būti integruojamas į klinikinę praktiką, taip prisidedant prie pacientų komforto, emocinės gerovės ir bendro pasitenkinimo procedūra.

## Introduction

Procedural pain and anxiety in interventional procedures are closely linked to both physiological and psychological aspects. Anxiety can enhance pain perception through attentional and emotional pathways, and inadequate pain control can increase intraoperative stress and provoke haemodynamic fluctuations [1]. Although standard sedation with midazolam or opioids is considered effective, it carries the risk of respiratory depression, prolonged recovery, and pharmacological interactions between drugs, especially in outpatient settings. Therefore, more interventional procedures are now performed with local anaesthesia alone, which is often insufficient to ensure maximum patient comfort [2,3].

Non-pharmacological approaches that reduce patient discomfort without additional sedation have become an area of active research. One of the interventions under investigation is immersive VR, which uses a computer-generated interactive environment to modulate the user’s visual and auditory senses, thereby influencing the subjective experience of pain and anxiety [4]. The therapeutic potential of VR is grounded in pain-gate control theory and cognitive-affective models of pain perception [5–7].

The beneficial analgesic and anxiolytic effects of VR during various procedures are increasingly being confirmed. Most interventions use special head-mounted displays that provide immersive, interactive, or passive audiovisual content to divert the patient’s attention from painful procedures. VR is effective in the treatment of acute burns, dental procedures, venipuncture in children, childbirth, and gastrointestinal endoscopy [8–12].

In a meta-analysis of 92 randomised clinical trials, Jhia J. Teh and co-authors showed that the use of VR in venepuncture-related, minimally invasive dressing burn procedures, and during childbirth significantly reduced subjective pain scores (*p* <0.01) and anxiety (*p* <0.01) [13].

Existing research on the use of VR is highly heterogeneous in terms of the nature of the VR content, the duration of the intervention, the methods used to evaluate the outcomes, and the methodological quality of the studies. Factors related to patients’ tolerance to virtual reality, such as cybersickness (a condition characterised by nausea, dizziness, and visual and vestibular discomfort), as well as the integration of VR technologies into the normal clinical workflow and the associated human and financial resources, remain under-researched. Therefore, further methodologically sound research is needed to provide a more robust assessment of the effectiveness, safety, and practicability of VR.

**The aim of this study** is to review the literature on the effectiveness of virtual reality in reducing procedural pain and anxiety among patients of different ages.

## Material and Methods

This manuscript was conducted as a narrative literature review. A literature search was performed in *PubMed, ScienceDirect, Web of Science*, and *Google Scholar* databases by using combinations of the following keywords: *virtual reality, immersive VR, head-mounted display, virtual environment, procedural pain*, and *anxiety*.

Articles published between January 2020 and January 2025 were considered. We included studies that evaluated the use of virtual reality for reducing pain and/or anxiety during medical procedures, peri-procedural care, or perioperative care. In addition to efficacy, articles addressing age-related considerations, VR safety, and adverse effects were included.

Selected studies included primary clinical studies (randomised and non-randomised), prospective/retrospective observational studies, and review articles (systematic reviews, meta-analyses). Articles not relevant to procedural pain or anxiety management, as well as duplicate publications were excluded.

Titles and abstracts were screened for relevance, followed by full-text review. For each paper, the following aspects were extracted: study design, population/age group, clinical setting/procedure, VR intervention type (active/passive, immersive/non-immersive), comparator, outcomes, and adverse effects.

The findings were synthesised narratively and organized by mechanism of action, perioperative applications, age groups, adverse effects, and limitations. To improve clarity, evidence was interpreted according to the study type.

The initial search identified 318 records. After removal of duplicates (n=74), 244 titles and abstracts were screened for relevance. Full-text assessment was performed for 92 articles. Based on relevance to the topic, methodological clarity, and focus on procedural pain and/or anxiety reduction during interventional procedures, 40 articles were included in this review.

**Figure 1:**
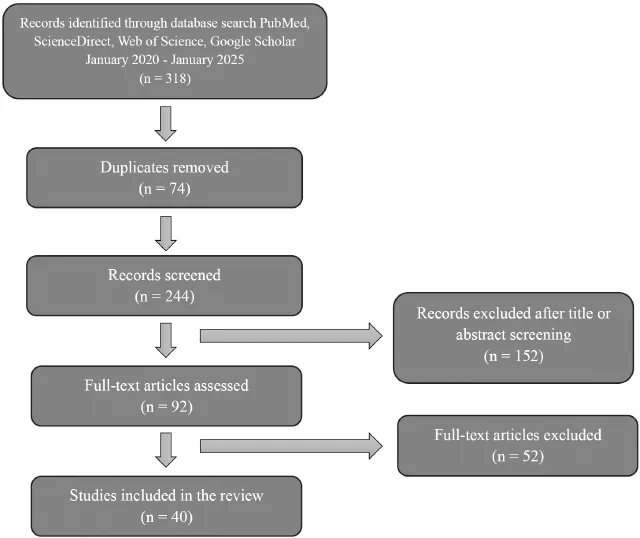
Flow chart of study selection

## Results

### The mechanism of action of VR in pain and anxiety relief

Virtual reality (VR) has gained a lot of attention in recent years as a safe tool to reduce procedural pain, anxiety, and stress for patients. VR is used for both interventional and minimally invasive or surgical procedures performed while the patient is conscious. As the method becomes more popular, it is important to understand its mechanism of action. Virtual reality reduces pain and anxiety by interacting with multiple levels of the body’s systems. The sensory system captures visual, auditory, and proprioceptive stimuli, diverting attention away from the procedure and reducing pain perception [4]. The nervous system, including the spinal cord’s pain-gating mechanism, modulates the transmission of pain signals to the cerebral cortex, and functional studies show reduced activity in the anterior cingulate cortex and insula, which are responsible for the emotional component of pain [6,7]. A decrease in sympathetic nervous system activity is reflected in reduced physiological stress indicators, such as the heart rate and blood pressure. Finally, the cognitive and affective systems direct attention, provide a sense of control, and reduce anxiety [14].

Virtual reality (VR) reduces anxiety through a combination of exposure-based learning, neurophysiological regulation, and attentional engagement. In VR exposure therapy (VRET), controlled virtual environments elicit psychophysiological arousal (e.g., electrodermal activity), thereby enabling habituation or extinction of fear responses [15]. Neuroimaging (fNIRS) studies in social anxiety disorder show that VR treatment leads to reduced activation in the frontopolar prefrontal cortex and orbitofrontal cortex, suggesting strengthened top-down regulation of emotional responses [16]. VR can also facilitate mindfulness-like states: brief VR mindfulness (nature-scene) interventions have been shown to decrease self-reported anxiety and increase alpha-band EEG power while reducing high-frequency beta activity in the anterior cingulate cortex [17]. Moreover, biofeedback embedded in VR can amplify anxiety reduction by modulating heart rate and autonomic function, providing users with real-time physiological feedback [18].

The integrated action of these systems forms a multi-layered mechanism that allows VR to effectively reduce procedural pain and improve the patient’s emotional state.

### VR applications in the periprocedural and perioperative period

Mainly in the paediatric population, multiple randomised controlled trials have shown that VR significantly reduces pain and anxiety compared to usual care or other distraction measures such as a kaleidoscope or ball pressure during interventions such as vaccination, blood draws, or intravenous catheter insertion [19–26]. The scales used by the researchers (Child Anxiety Scale, Child Fear Scale, and others) have shown scores ranging from 30.8% to 53.3% for pain and 52% for anxiety reduction [20,24]. In addition to sensory stimulation, the VR environment acts as an interactive experience, allowing the patient to focus on alternative activities and to be more subjectively in control of the situation during the procedure. A multicentre feasibility randomised controlled trial revealed that it is associated with greater confidence, fewer negative emotional reactions, and a more positive assessment of the overall procedural experience [27].

The level of VR interactivity can affect the intervention’s effectiveness. Studies distinguish between passive forms of VR, in which the patient observes soothing natural images or short animated scenes about the procedure, and active forms of VR that require interaction with the virtual environment, such as interactive games. Reductions in pain and anxiety were observed in both cases, but active VR programmes were more often associated with more pronounced emotional relaxation and lower estimates of distress [7–8,28]. According to a systematic review and meta-analysis, VR tools are generally well tolerated during interventional procedures and only occasionally cause mild, transient adverse events, such as nausea or dizziness [29]. Studies also highlight the additional benefits of VR preoperatively: patients have a clearer understanding of the procedure, its goals, and possible consequences when shown a realistic 360° operating room environment and when operating room sounds are integrated before the procedure. Virtual simulation helps to reduce anxiety related to the unknown waiting procedure [30,31]. Besides that, one of the most critical findings was a significant reduction in emotional exhaustion among nurses who used VR for patient education. This reduction is particularly important, as burnout remains a pressing issue in nursing, especially in high-stress environments like surgical wards [30,32].

In the obstetrics and gynaecology context, the use of VR during labour, including episiotomy, has been associated with a reduction in pain intensity on the VAS scale from 2.6 pre-intervention to 0.8 post-intervention, and with a more positive birth experience as perceived by the mother [33,34].

In orthopaedic and endovascular procedures, VR helps distract from nociceptive stimuli, reduces the need for analgesics, and lowers physiological stress indicators such as the heart rate and arterial blood pressure [35,36]. In dentistry and oral surgery, short VR sessions reduce pre-procedural anxiety, improve the patient comfort, and may reduce the need for pharmacological sedation [37], while in cardiology interventions, the use of VR before or during the procedure has been associated with significantly lower levels of anxiety and higher patient satisfaction [38].

Evidence reported in the literature reviews shows that VR interventions are effective in reducing pre- and intra-operative anxiety in both children and adults. The mechanism involves shifting attention away from the procedure and reducing sympathetic nervous system activity, thereby reducing physiological indicators of stress [31,39–40]. It is important to note that patients with high baseline levels of anxiety may be slightly less affected, and there is a lack of research on long-term effects [41–43]. In children and adolescents as well as adults, virtual reality interventions can significantly reduce pre-procedural and intra-procedural anxiety during surgical procedures. According to findings from recent randomised clinical studies, in the adult population, educational and relaxation applications displayed in VR reduce the level of preoperative anxiety before elective surgical and invasive cardiac or endovascular procedures and improve patient experience and satisfaction with treatment [33–38].

In the paediatric and adolescent group, VR was most used as a distraction for needle-related procedures (vaccination, peripheral vein puncture, and blood sampling). Studies have shown that VR reduces procedural anxiety and fear in children, and that parents’ evaluation of the procedure is more positive [19,21]. In addition to subjective anxiety scales, vital signs were also assessed, but their changes were less consistent and did not always match the dynamics of subjective anxiety [20,22]. The results suggest that the effect of VR may be lower in patients with very high baseline levels of anxiety or specific clinical characteristics, which suggests that individual patient factors may modify the effectiveness of the intervention [40].

### VR applications in different age groups

Virtual reality (VR) has demonstrated efficacy in reducing procedural pain, anxiety, and promoting engagement across the lifespan, but tolerability and safety are strongly age-dependent. In paediatric populations, systematic reviews and meta-analyses report significant reductions in procedural pain and anxiety, with transient adverse effects such as nausea, dizziness, and visual discomfort occurring in approximately 3–12% of sessions, and, although manufacturers typically recommend a minimum age of 10–13 years, clinical studies document safe use in children as young as 4–7 years when sessions are brief (≤10 min), content is age-appropriate, and supervision ensures proper headset fit and adherence to stop protocols [44–46]. Systematic reviews and meta-analyses have indicated that, in adults, VR is widely used for peri-procedural distraction, mental health interventions, and rehabilitation, with adverse events primarily driven by device latency, motion intensity, or vestibular susceptibility rather than chronological age, and tolerability rates generally exceeding 85% in clinical trials [47–48]. In older adults, VR can improve mood, engagement, and cognitive function, including in mild cognitive impairment. Still, frailty, vestibular sensitivity, sensory deficits, and cognitive decline increase the risk of cybersickness, imbalance, and disorientation. Systematic reviews recommend seated, low-motion experiences, short supervised sessions, and pre-screening for vestibular disorders, photosensitive epilepsy, and the patient’s ability to follow instructions [49,50]. Patients with moderate–severe dementia or non-communicative individuals require individualized risk–benefit assessment because VR may provoke confusion or distress, though structured, simplified environments can still provide engagement and affective benefit under caregiver supervision [51]. Collectively, these findings indicate that VR protocols should be tailored to developmental and functional status rather than chronological age alone, optimizing the session duration, content complexity, and supervision to maximize efficacy and minimize adverse effects across the lifespan.

### Safety and potential adverse effects

Virtual reality (VR) is generally well tolerated in clinical settings, and most reported adverse effects are mild, transient, and self-limited. Adverse effects vary by age, health status, and device characteristics, session duration, and content design [44,52,54]. In children, transient symptoms such as nausea, dizziness, eye strain, and diplopia have been reported, particularly with prolonged sessions or improperly fitted headsets, though serious events are rare [44,52]. Adults may experience cybersickness related to motion mismatch, latency, or high visual flow, with prevalence estimates ranging from 5–20% depending on the content and exposure time [54]. In older adults, frailty, vestibular sensitivity, sensory deficits, and cognitive impairment increase susceptibility to imbalance, disorientation, and nausea, thereby making short, seated, low-motion sessions with supervision and pre-screening recommended [55,56]. Photosensitive epilepsy is a rare but recognized contraindication, and all users should have the ability to communicate discomfort and follow stop instructions [57,58]. Careful session design, monitoring, and individualized risk assessment are essential to maximize safety across all age groups.

### Outcome heterogeneity and practical implementation considerations

An important consideration when interpreting the current evidence is the heterogeneity of outcome measures and intervention protocols across studies. Pain and anxiety were assessed by using various scales at different procedural time points and across different clinical contexts [19–26]. Additionally, VR interventions varied in content (passive/interactive), immersion level, session duration, and comparator conditions (usual care/other distraction methods). This heterogeneity limits direct comparisons of the observed effects and reduces the researcher’s ability to draw conclusions about a single optimal protocol for VR use, even when the overall result is positive [33–40].

For implementation in clinical care, several practical factors should be considered. The cost may include headset purchase, software licensing, accessory replacement, and staff time for setup and supervision [30,32]. Staff training is needed to ensure appropriate patient selection, device fitting, troubleshooting, monitoring for adverse effects, and integration into the clinical practice without delaying patient care [49–52].

Implementation of clinical VR has been described in paediatric hospital practice where VR is integrated into routine supportive care workflows and delivered by trained staff in the *Stanford Chariot* program [52]. Institutional standard operating procedures highlight the importance of staff onboarding, including patient screening, brief orientation, and establishment of a stop-signal, proper device fitting, monitoring for adverse effects, and documentation of VR use [59]. To prevent infection, guidance recommends cleaning and disinfecting VR headsets after each use, performing hand hygiene, using disposable or washable face covers, allowing devices to dry properly, and following local infection control policies and manufacturer instructions [60]. Overall, addressing these implementation factors may improve the feasibility, safety, and sustainability of VR adoption in routine procedural care.

## Limitations of the study

Most of the studies reviewed have small sample sizes and use heterogeneous methodologies, thus limiting the ability to determine the impact of virtual reality on the population. It is important to emphasize the need for more long-term follow-up studies evaluating the impact of VR interventions in repeated interventions. Additionally, heterogeneity in outcome measures, assessment timing, VR content, and comparator interventions limited direct cross-study comparability and precluded identification of a single standardized protocol.

## Conclusions

The literature analysis shows that VR is a promising tool for reducing procedural pain and anxiety. VR is an effective non-pharmacological tool for patients of all ages and is safe for different interventions. The integration of VR provides both physical comfort and educational benefits, improving patients’ emotional state and the overall satisfaction with the procedure. Future studies should aim to standardize VR protocols, evaluate long-term effects, and analyse methodological differences to more clearly determine the effectiveness of VR interventions in different clinical settings.
